# Hepatoprotective Effect of *Antrodia camphorata* Mycelium Powder on Alcohol-Induced Liver Damage

**DOI:** 10.3390/nu16193406

**Published:** 2024-10-08

**Authors:** Unyong Kim, Sung-Il Jang, Pei-Ni Chen, Shingo Horii, Wu-Che Wen

**Affiliations:** 1 Division of Bioanalysis, Biocomplete Inc., #603, 604, Hanshin IT Tower, 272 Digital-ro, Guro-gu, Seoul 08389, Republic of Korea; 2 Golden Biotechnology Corporation, Tamsui District, New Taipei City 251, Taiwan; sijang@goldenbiotechnology.co.kr (S.-I.J.); pnchen79@goldenbiotech.com (P.-N.C.); shin1822@goldenbiotech.com (S.H.); wwc@goldenbiotech.com (W.-C.W.)

**Keywords:** *A. camphorata*, liver steatosis, hepatoprotectants

## Abstract

Background/Objectives: *Antrodia camphorata*, also known as “Niuchangchih” in Taiwan, is a unique medicinal mushroom native to Taiwan. It is used in traditional medicine to treat various health conditions. In this study, we investigated the efficacy of *A. camphorata* mycelia on alcohol-induced liver damage, both in vitro and in vivo, in a Good Laboratory Practice (GLP) facility. Methods: The experimental groups consisted of a normal control group (G1), a negative control group (G2), an *A. camphorata* mycelium powder 50 mg/kg/day administration group (G3), a 100 mg/kg/day administration group (G4), a 200 mg/kg/day administration group (G5), and a positive control silymarin 200 mg/kg/day administration group (G6), with 10 Sprague Dawley rats assigned to each treatment group. Results: We found that treatment with *A. camphorata* mycelium powder significantly reduced alanine aminotransferase, aspartate aminotransferase, alkaline phosphatase, cholesterol, adiponectin, triglyceride, and malondialdehyde concentrations. Histopathological analysis also revealed that the inflammation score significantly decreased in the *A. camphorata*-treated groups. Conclusion: Based on these results, we conclude that repeated oral administration of *A. camphorata* mycelium powder is effective in improving alcoholic liver disease.

## 1. Introduction

Chronic and excessive alcohol intake significantly contribute to the development of various diseases, such as diabetes mellitus, hypertension, dementia, and depression [1]. In addition, alcohol mainly causes liver injury and results in alcoholic liver disease (ALD) because the liver serves as the principal organ involved in ethanol metabolism [2]. Reactive oxygen species (ROS) are produced during ethanol metabolism. ROS causes hepatic damage and may also lead to vascular injury, which may contribute to the development of various diseases [3,4,5]. Depending on the various factors affecting disease development, such as genetic variants, sex, obesity, ethnicity, and viral hepatitis, these diseases range from alcoholic steatosis to steatohepatitis, fibrosis, and even cirrhosis [3,6,7]. These liver diseases predominantly affect middle-aged and elderly individuals, who play significant social and economic roles. This leads to substantial social and economic losses that exceed those caused by smoking and obesity [8]. Consequently, there is an immediate necessity to develop new therapeutic and preventive agents against liver diseases.

*Antrodia camphorata*, also known as “Niuchangchih” in Taiwan, is a unique medicinal mushroom native to Taiwan. It has been used as a traditional medicine to treat various health-related conditions because it shows various pharmacological activities, ranging from immunomodulatory to anti-cancer effects [9,10,11]. In particular, the fruiting bodies of *A. camphorata* have historically been used to relieve symptoms associated with alcohol consumption in Taiwan [9,11]. It is also used as a traditional medicine or dietary supplement for treating various diseases, such as cancer, diarrhea, liver injury, inflammation, abdominal pain, hypertension, itchy skin, and motion sickness [10,11]. However, it has a very slow growth rate and is hardly noticeable until the host tree enters the wild [9]. Moreover, the cultivation and development of fruiting bodies of *A. camphorata* present significant challenges, even within a controlled laboratory environment [10]. Therefore, numerous cultivation methods have been devised to meet its increasing market demand [10,12].

*A. camphorata* contains a variety of biologically and pharmacologically active components, with terpenoids, including antroguinonol, being the primary component [10]. Antroquinonol, one of the major components in A. camphorata, exerts a wide variety of pharmacological effects. For instance, antroquinonol suppresses the ERK-AP-1 and AKT-NFκB pathway and then prevents migration and invasion of cancer cells [13]. In addition to the anti-cancer activity, it can reduce cellular oxidative stress by inducing the Nrf2 signaling pathway, which promotes the expression of heme oxygenase-1 and inhibits the production of free radicals, as well as increases the level of GSH [13].

Extensive research has been accomplished to evaluate the efficacy of *A. camphorata* on various diseases; however, little research has been conducted by institutions that meet Good Laboratory Practice (GLP) regulations. In this study, we conducted in vitro and in vivo experiments to investigate the hepatoprotective effects of *A. camphorate* mycelia using an ethanol-intoxicated cell line and rats as an experimental model in a GLP facility. We evaluated the clinical symptoms, body weights, blood biochemical analysis, γ-GTP, adiponectin, and liver weights and performed liver tissue and histopathological analyses in vivo.

## 2. Materials and Methods

*A. camphorata* mycelium powder produced by submerged culture was provided by Golden Biotechnology (New Taipei City, Taiwan), and it contains antroquinonol as the major pharmacological component at a concentration of 15.17 mg/g. The content of antroquinonol was determined by HPLC and conducted by Golden Biotechnology. To evaluate the efficacy of *A. camphorata* mycelium powder on alcohol-induced liver damage, in vitro and in vivo studies were designed and conducted under GLP conditions.

### 2.1. Cell Lines and Cell Culture Conditions

The human liver cancer cell line HepG2 was obtained from the Korean Cell Line Bank (Seoul, Republic of Korea). HepG2 cells were cultured in serum-free Dulbecco’s modified Eagle’s medium (DMEM) supplemented with 10% fetal bovine serum and antibiotics (100 U/mL of penicillin and streptomycin) at 37 °C in 5% CO_2_. The cells were then subcultured in complete DMEM and seeded in each well of 96 well plates at appropriate cell numbers (ranging from 5 × 10^4^ to 3 × 10^5^ cells) for the assays. The cells were incubated for 24 h at 37 °C in 5% CO_2_, and then the spent culture medium was removed before administering the experimental treatments.

### 2.2. Cytotoxicity Assay

*A. camphorata* mycelium powder was dissolved in phosphate-buffered saline (PBS) to generate a 1000 µg/mL solution. Subsequently, a two-fold serial dilution was applied, resulting in a final dose of the test substances at a concentration of 0.977 µg/mL in serum-free DMEM. For the experimental treatment of the test substances, 1 × 10^4^ cells were inoculated per well of 96-well plates and incubated for 24 h. The spent medium was then removed and replaced with 200 µL of the test substances with different concentrations. Negative control cells were treated with serum-free DMEM. After the treatment, the spent culture media containing the test substances were removed from each well and replaced with 200 µL of serum-free DMEM. Then, 20 µL of WST-1 solution was added to each well. The cells were incubated for 2 h at 37 °C in 5% CO_2_, and the optical density (OD) at 450 nm was measured using a microplate reader (SpectraMAX PLUS, Molecular Device, San Jose, CA, USA). Cell viability was calculated as the percentage ratio of the OD of the treated cells relative to that of the negative control cells. The inhibition rate was calculated using the following formula:

Inhibition rate (%) = [1 − (B − A)/B] × 100

where A is the mean OD of the wells treated with each dose of the test substance, and B is the mean OD of the wells treated with the negative control. When cell viability was reduced, the 50% inhibitory concentration (IC_50_) was calculated and evaluated using regression analysis.

### 2.3. In Vitro Efficacy Assays

For the in vitro efficacy assays, 1.2 × 10^5^ cells for superoxide dismutase (SOD) activity and glutathione S-transferase (GST) assays or 1.0 × 10^4^ cells for alanine aminotransferase (ALT) and aspartate aminotransferase (AST) assays were seeded into each well of 24-well plates and cultured in complete DMEM at 37 °C and 5% CO_2_ for 24 h. The spent medium was then removed and replaced with 200 µL of serum-free DMEM containing different concentrations of the test substance (1, 4, and 16 µg/mL), the vehicle (serum-free DMEM) as the negative control, or silymarin (25 µM) as the positive control. Each treatment was conducted in triplicate. The cells were incubated at 37 °C for 1 h in 5% CO_2_ and then exposed to 200 mM ethanol for 23 h. Superoxide dismutase activity, GST activity, ALT, and AST assays were carried out using cell lysates according to the manufacturer’s instructions.

### 2.4. Oil Red O (ORO) Staining

For ORO staining, 2.0 × 10^4^ cells were seeded per well of 24-well plates and incubated for 24 h in a humidified incubator at 37 °C and 5% CO_2_. The spent medium was then removed and replaced with 200 μL of fresh medium containing different concentrations of the test substance (1, 4, and 16 µg/mL). The negative control group received a complete culture medium only. Each treatment was conducted in triplicate. The cells were incubated for another hour and then exposed to 200 μL of 300 mM palmitic acid solution for 23 h. After palmitic acid exposure, the cells were fixed with 4% paraformaldehyde for 30 min and washed with PBS. The cells were stained with an ORO solution for 30 min at room temperature (15~25 °C) and washed with PBS. The stained cells were extracted with 100% isopropanol and incubated at room temperature for 5 min. The supernatants were transferred to 96-well plates, and the absorbance was measured at 510 nm using a microplate reader (SpectraMAX PLUS, Molecular Devices, San Jose, CA, USA). The lipid content was calculated as the percentage of absorbance of the treated cells relative to that of the negative control cells.

### 2.5. Animal Studies

A 6-week repeated oral dose study was performed to evaluate the effect of *A. camphorata* mycelium powder on alcoholic fatty liver in Sprague Dawley rats. This study was approved by the Institutional Animal Care and Use Committee (IACUC) of the Nonclinical Research Institute, CORESTEMCHEMON Inc. (Serial No. 2023-0081, approved on 21 March 2023). The experimental groups consisted of a normal control group (G1); a negative control group (G2); groups treated with 50 mg/kg/day *A. camphorata* (G3), 100 mg/kg/day *A. camphorata* (G4), and 200 mg/kg/day *A. camphorata* (G5); and a positive control group treated with 200 mg/kg/day silymarin (G6). Ten rats were assigned to each treatment group (Table 1). Detailed information on each group is summarized in Table 1. The animals were housed in a room that was maintained at a temperature of 22 ± 3 °C, humidity of 55 ± 15%, 12 h light/12 h dark cycle (from 08:00 to 20:00), 150–300 Lux of luminous intensity, and 10–20 air changes per hour. Ethanol was fed to Sprague Dawley rats in groups G2–G6 to induce alcoholic fatty liver. The normal control group (G1) was fed a Lieber–DeCarli regular control liquid diet (Dyets#710027, Dyets Inc., Bethlehem, PA, USA), and the alcoholic fatty liver AFL-induced groups (G2–G6) were fed a Lieber–DeCarli ethanol liquid diet (Dyets#71100260, Dyets Inc.) with ethanol (0.816 g/mL). For adaptation to ethanol, the alcoholic fatty liver (AFL)-induced groups were fed an ethanol liquid diet supplemented with 1% (*w*/*v*) ethanol for 3 days, followed by the addition of 3% (*w*/*v*) ethanol for another 4 days. Calorie loss due to ethanol content was supplemented with maltodextrin. After adaptation to ethanol, the animals were fed 5% (*w*/*v*) ethanol for six weeks. The liquid diet was prepared daily. The animals were orally administered 5 g/kg ethanol once every two weeks during the test article administration period.

Analyses of clinical symptoms, body weights, blood biochemical analysis, γ-GTP, adiponectin, liver weights, and liver tissue, along with histopathological analyses, were evaluated. Blood samples were collected once every two weeks. The animals were fasted for approximately 18 h before blood collection. Blood was left at room temperature to clot for approximately 30 min and centrifuged for 10 min at 3000 rpm to separate the serum. The sera were divided into two vials: one for blood chemical analysis and the other for analyzing γ-GTP and adiponectin levels.

### 2.6. Clinical Signs and Body Weight Monitoring

All the animals were examined daily for mortality and general symptoms during the administration and observation periods. Body weight was also measured upon receipt and grouping and twice a week during the administration period.

### 2.7. Blood Biochemical Analysis, γ-GTP, and Adiponectin Assays

Blood biochemical parameters were determined using a serum biochemical analyzer (AU680, Beckman Coulter, Brea, CA, USA). The parameters examined included aspartate aminotransferase (AST), alanine aminotransferase (ALT), alkaline phosphatase (ALP), glucose (GLU), triglyceride (TG), total cholesterol (CHO), low-density lipoprotein cholesterol (LDL), and high-density lipoprotein cholesterol (HDL). The serum concentrations of γ-GTP (MBS9343646, Mybiosource, San Diego, CA, USA) and adiponectin (RRP300, R&D system, Minneapolis, MN, USA) were measured using ELISA kits once every two weeks.

### 2.8. Liver Tissue Sampling and Analysis

On the day of necropsy, the animals were anesthetized with 3–5% isoflurane, and the maximum volume of blood samples was collected from the posterior vena cava using a syringe. The liver was carefully removed and weighed. After weighing, the liver was divided into the middle lobe and other lobes. Colorimetric and qPCR assays were conducted using the other lobes. The parameters for colorimetric assays were lipid peroxidation (MDA (TBARS), MAK085, Sigma-Aldrich, St. Louis, MO, USA), CuZn-superoxide dismutase (SOD, MBS036924, Mybiosource), TG, alcohol dehydrogenase (ADH, MBS700352, Mybiosource), acetaldehyde dehydrogenase (ALDH, MBS010987, Mybiosource). qPCR assays were performed for fatty acid synthase (FAS), *CYP2E1*, cannabinoid receptor 1 (CB1), and estrogen-related receptor-γ (ERR-γ).

### 2.9. Histopathological Analysis

The middle lobe of the liver was preserved in 10% formalin and divided into two sections. The first section was processed using an automated tissue processor for paraffin embedding to form a paraffin block. The block was sectioned into thin slices and stained with hematoxylin and eosin. The second section was cryoprotected by submerging it in 30% sucrose for three days at a temperature of 4 °C. This tissue was then embedded in an OCT compound to create a frozen block, which was sectioned into thin slices and stained with Oil Red O (ORO). The stained tissue slides from both sections were examined under a light microscope (BX61; Olympus, Tokyo, Japan). Images were captured using a camera (DP80, Olympus, Japan) and analyzed quantitatively using Image-Pro software (Ver 10, Media Cybernetics, Rockville, MD, USA).

### 2.10. Statistical Analysis

For the in vitro study, statistical analyses were performed using SPSS Statistics software (version 12.0) for Medical Science. Bartlett’s test was used to determine the homogeneity of the variance, with a significance level of 0.05. One-way analysis of variance (ANOVA) was performed to assess the homogeneity of the variance. If the ANOVA results were significant, Dunnett’s *t*-test was employed for multiple comparisons, setting the significance levels at 0.05 and 0.01 (one-tailed). For data that did not meet the homogeneity criteria, the Kruskal–Wallis test was employed. If the results were significant, Steel’s test was performed for multiple comparisons, setting the significance levels at 0.05 and 0.01 (one-tailed).

In the animal studies, parametric multiple comparison procedures were performed to analyze data across all groups. To confirm the induction of alcoholic fatty liver, we compared the normal control group (G1) and the negative control group (G2) using the Student’s *t*-test. When the variances were unequal, Welch’s *t*-test was applied. The negative control group (G2) and the administration groups (G3–G5) were compared to evaluate the effects of the test compound. In addition, the administration groups (G3–G5) and the positive control group (G6) were compared for the same purpose. To verify the safety of the increase in blood glucose caused by the test compound on week 6, we compared the normal control group (G1) and administration groups (G3–G5). One-way ANOVA was used to compare groups. If the results were significant, Duncan’s test for equal variances and Dunnett’s test for unequal variances were conducted for post hoc testing, with the significance level set at 0.05.

## 3. Results

### 3.1. Cytotoxicity Assay

**Figure 1:**
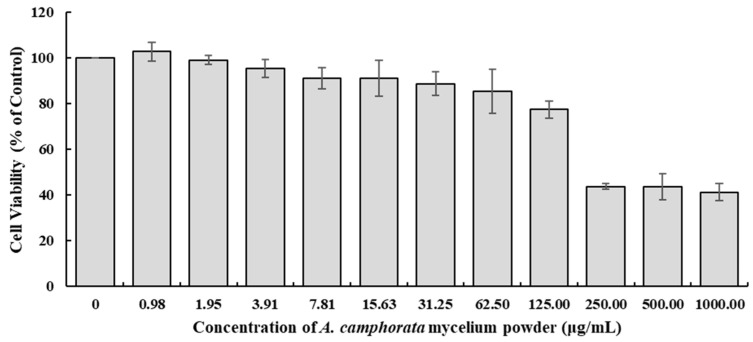
Cytotoxicity of *A. camphorata* mycelium powder.

The HepG2 cell line has been widely used to model fatty liver diseases, including non-alcoholic and alcoholic fatty liver diseases [14,15]. The HepG2 cell line retains a variety of metabolic functions of human hepatocytes, such as lipid metabolism, which is crucial for studying fatty liver disease. In addition, it can accumulate lipids when exposed to free fatty acids or ethanol, mimicking the steatosis seen in non-alcoholic fatty liver diseases (NAFLD) and alcoholic fatty liver disease (AFLD). Therefore, we selected the HepG2 cell line to evaluate the in vitro efficacy of *A. camphorata* mycelium powder against alcohol-induced fatty liver disease. However, many research studies have reported that *A. camphorata* is cytotoxic to carcinoma cells. Therefore, selecting the appropriate dose is crucial for the accurate interpretation of the results. To select the appropriate doses for the in vitro studies, the cytotoxicity of the *A. camphorata* mycelium powder was evaluated at the concentration range from 0.977 µg/mL to 1000 µg/mL. As shown in Figure 1, the cell viability in the treated groups decreased compared to the non-treated group in a dose-dependent manner; the calculated IC_50_ was 187 µg/mL. Based on the cell viability results, the doses of the *A. camphorata* mycelium powder were selected at 0, 4, and 16 µg/mL for the in vitro studies, which elicited cell viabilities of over 90% compared with the non-treated control.

### 3.2. In Vitro Activity Assays

To evaluate the in vitro efficacy of *A. camphorata* mycelium powder on alcoholic fatty liver disease (AFLD), an alcoholic fatty liver model was established by treating HepG2 cells, a human liver cancer-derived cell line, with 200 mM ethanol. A series of in vitro activity assays were performed after the induction of the AFLD model. The results of each in vitro assay are presented in Table 2.

A superoxide dismutase (SOD) activity assay was conducted to evaluate liver protection via the clearance of ROS produced by the alcoholic fatty liver. When the SOD activity of the normal control was considered 100%, the average SOD activity in each group ranged from 90.6% to 170.5%. Although the estimated SOD activities were not statistically significant, SOD activity tended to increase in the groups treated with *A. camphorata* mycelium powder and in the positive control (silymarin-treated group), compared with the negative control. The highest SOD activity was observed in cells treated with 4 μg/mL *A. camphorata* mycelium powder.

GST activity was evaluated after treatment with *A. camphorata* mycelium powder concentrations of 0, 1, 4, and 16 µg/mL and 25 µM silymarin as a positive control. When the GST activity of the normal control was considered 100%, the average GST activity in each group ranged from 82.8% to 119.6%. The GST activities in the treated groups were significantly higher compared with those of the negative control. These results showed that *A. camphorata* mycelium powder protected the liver from ROS by increasing antioxidant defense mechanisms [16,17].

ALT and AST assays were also performed to evaluate the protective effects of *A. camphorata* against liver injury. ALT concentrations in the normal control, negative control, positive control, and test groups ranged from 674.3 to 728.1 µg/mL. ALT levels were similar between the negative control group and the normal control group, and there was no difference between the negative control group, the *A. camphorata* mycelium powder-treated groups, and the positive control group. On the other hand, in the case of AST, significant changes were observed in both test substance-treated groups (181.1, 171.5, and 155.1 pg/mL for the 1, 4, and 16 µg/mL-treated groups, respectively) and the positive control group (157.0 pg/mL) compared with the negative control group (198.5 ± 11.8 pg/mL). The concentration of AST in the negative control group was significantly higher than that in the normal control group (155.1 pg/mL) and decreased in an *A. camphorata* dose-dependent manner in the test substance-treated group.

An ORO staining assay was used to evaluate the lipid content in the cells. The observed OD values observed at 550 nm ranged from 0.044 to 0.221. The negative control group exhibited significantly higher lipid content compared to the positive control group, indicating lipid accumulation. The OD values significantly decreased in an *A. camphorata* dose-dependent manner compared to the negative control.

Taken together, we hypothesized that *A. camphorata* mycelium powder could exert hepatoprotective effects by reducing lipid accumulation and scavenging ROS by increasing GST activity.

### 3.3. In Vivo Studies

#### 3.3.1. Clinical Signs and Body Weight

**Figure 2:**
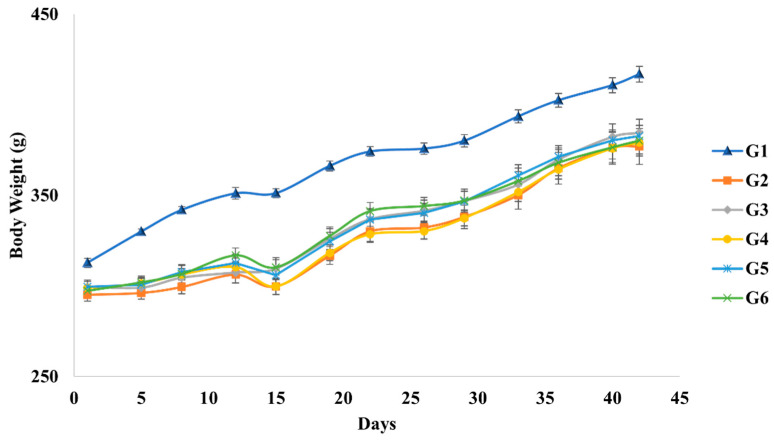
Body weight changes of each group during the experimental period.

We monitored both the clinical signs and body weight of rats to determine the toxic effects of *A. camphorata* mycelium powder. A significant reduction in body weight was observed in the negative control group (G2) throughout this study compared to the normal control group (G1) (*p* < 0.01). The body weights of the groups induced with alcoholic fatty liver (G2–G6) did not differ significantly (Figure 2). In addition, we did not observe any toxicity-related abnormalities during the experimental period.

#### 3.3.2. Blood Biochemical Analysis

The blood biochemical analysis, γ-GTP, and adiponectin assays were conducted in six groups of rats: G1 (normal control), G2 (negative control), G3–G5 (test groups treated with different doses of *A. camphorata* mycelium powder), and G6 (positive control).

**Figure 3:**
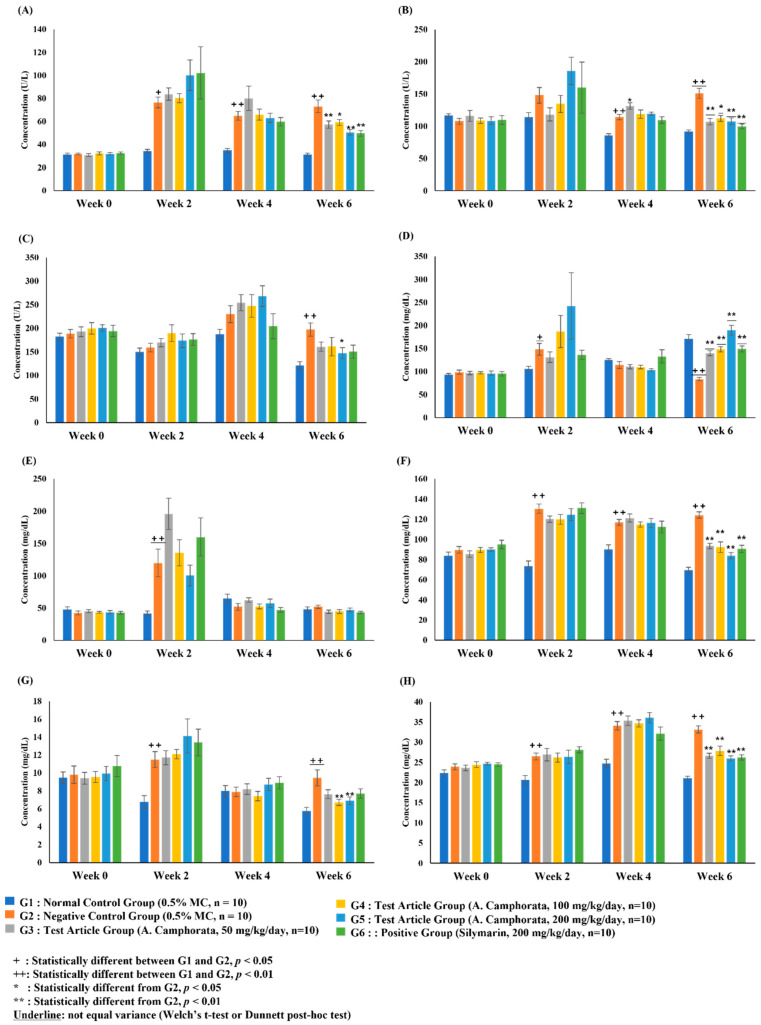
Concentration of biochemical markers in rat serum: alanine aminotransferase (**A**), aspartate aminotransferase (**B**), alkaline phosphatase (**C**), glucose (**D**), triglycerides (**E**), cholesterol (**F**), low-density lipoprotein (**G**), and high-density lipoprotein (**H**). Data are presented as the mean ± SE. The results were statistically analyzed using Student’s *t*-test or one-way ANOVA.

The ALT level in the negative control group significantly increased after week 2 (*p* < 0.01) compared to that in the control group. There were no significant differences in ALT levels between the other test substance administration groups and the negative control group at weeks 0, 2, and 4. In contrast, on week 6, the ALT level in the *A. camphorata* mycelium powder administration groups was significantly lower than that in the negative control group (*p* < 0.05). ALT levels in the positive control group were also significantly lower than those in the negative control group on week 6 (*p* < 0.01) (Figure 3A).

The AST level in the negative control group significantly increased after week 2 (*p* < 0.01) compared to that in the control group. There were no significant differences in AST levels between the other test substance administration groups and the negative control group at weeks 0, 2, and 4. In contrast, on week 6, the AST level in the *A. camphorata* mycelium powder administration groups was significantly lower than that in the negative control group (*p* < 0.01). Similarly, the AST level in the positive control group was significantly lower than that in the negative control group at week 6 (*p* < 0.01) (Figure 3B).

The ALP level in the negative control group was significantly increased on week 6 (*p* < 0.01) compared to that in the control group. None of the *A. camphorate*-treated groups showed a significant difference in ALP levels compared with that of the negative control in weeks 0, 2, and 4. In contrast, on week 6, the ALP level of the *A. camphorata* mycelium powder 200 mg/kg/day administration group was significantly lower than that of the negative control (*p* < 0.05). In addition, the AST level in the positive control group was significantly lower than that in the negative control on week 6 (*p* < 0.05) (Figure 3C).

The blood GLU level in the negative control group was significantly increased in week 2 (*p* < 0.05) and markedly decreased in week 6 (*p* < 0.01) compared with that in the normal control group. There were no significant differences in GLU levels between the test substance administration groups and the negative control group at weeks 0, 2, and 4. In contrast, on week 6, the GLU level in the *A. camphorata* mycelium powder administration groups was significantly higher than that in the negative control group (*p* < 0.01). None of the groups treated with *A. camphorata* mycelium powder showed significant differences in GLU levels compared to the silymarin-treated group (Figure 3D).

The TG levels in the negative control group were significantly higher than those in the normal control group on week 2 (*p* < 0.01). The TG level in the 50 mg/kg/day *A. camphorata* mycelium powder administration group was significantly higher than that in the negative control at week 2 (*p* < 0.05). Other groups administered *A. camphorata* mycelium powder did not show statistically significant differences compared with the negative control group at week 2. The TG level in the positive control group was significantly lower than that in the negative control at week 6 (*p* < 0.01). However, none of the *A. camphorata* mycelium powder-treated groups showed a statistically significant difference in TG levels compared to the positive control group (Figure 3E).

The blood CHO level in the negative control group significantly increased after week 2 compared to that in the control group (*p* < 0.01). CHO levels in the *A. camphorata* mycelium powder-treated groups and the positive control group were significantly decreased compared to the negative control group on week 6 (*p* < 0.01). However, none of the groups treated with *A. camphorata* mycelium powder showed significant differences in CHO levels compared to the silymarin-treated group (Figure 3F).

The LDL levels in the negative control group were significantly higher than those in the normal control group on weeks 2 and 6 (*p* < 0.01). On week 6, significant changes in LDL levels were observed in G4 and G5 compared to those in the negative control group (Figure 3G). Similarly, HDL levels in the negative control group were significantly higher than those in the normal control group after week 2 (*p* < 0.01). All *A. camphorata* mycelium powder-treated groups and the positive control group showed a statistically significant decrease in HDL levels compared to the negative control group on week 6 (*p* < 0.01). None of the *A. camphorata* mycelium powder-administered groups showed a statistically significant difference in HDL levels compared to the positive control group (Figure 3H).

#### 3.3.3. γ-GTP and Adiponectin Assay

There was no significant difference in the γ-GTP level among all groups. However, during weeks 2 and 6, the serum γ-GTP level in the negative control group increased by more than 85% compared to the normal control group. Although we did not observe statistically significant changes in γ-GTP levels, a reduction in γ-GTP levels was observed in all groups treated with *A. camphorata* mycelium powder compared to the negative control group. Specifically, the γ-GTP level decreased more than 12.3% at week 2, 19.4% at week 4, and 24.3% at week 6 compared to the negative control.

Adiponectin levels in the negative control group were significantly higher than those in the normal control group on week 2 (*p* < 0.01). On week 6, the adiponectin level was significantly decreased in the groups treated with 50 and 100 mg/kg/day of *A. camphorata* mycelium powder compared to that in the negative control group. Similarly, the positive control group exhibited significantly lower levels than the negative control group. However, adiponectin levels in the group treated with 200 mg/kg/day of *A. camphorata* mycelium powder did not differ significantly from the negative or the positive control groups.

#### 3.3.4. Liver Tissue Analysis

There was no statistically significant difference in liver weight among all experimental groups. In contrast, the liver-to-body weight ratio of the negative control group was significantly higher than that of the normal control group (*p* < 0.01). There was no statistically significant difference in the liver weight-to-body weight ratio in any of the AFL-induced groups (G2–G6) (Table 3).

The malondialdehyde (MDA) levels in the negative control group were significantly higher than those in the normal control group (*p* < 0.01). All *A. camphorata* mycelium powder-administered groups exhibited significantly decreased MDA levels compared to the negative control group (*p* < 0.01). However, we did not observe significant differences in the MDA levels between the positive and the negative control groups (Table 4).

Liver TG levels were significantly higher in the negative control group than in the normal control group. We observed significantly lower TG levels in the *A. camphorata* mycelium powder-administered groups at doses of 100 and 200 mg/kg/day than in the negative control group (*p* < 0.05). Additionally, the TG levels in G4 and G5 did not exhibit statistically significant differences compared to those in the positive control. However, the group administered *A. camphorata* mycelium powder at a dose of 50 mg/kg/day showed a significantly higher TG level compared to the positive control (*p* < 0.01) (Table 4).

The levels of SOD, alcohol dehydrogenase (ADH), and aldehyde dehydrogenase (ALDH) did not show any statistically significant differences across all the experimental groups (Table 4). Additionally, there were no statistically significant differences in the expression levels of proteins, including FAS, CYP2E1, CB1, and ERRγ, among all the experimental groups (Table 5).

#### 3.3.5. Histopathological Analysis

The steatosis score in the negative control group was significantly higher than that in the normal control group (*p* < 0.01). Although we did not observe any statistical differences among the groups, we identified the potential for improving steatosis in the groups treated with *A. camphorata* mycelium powder at doses of 100 (G4) and 200 mg/kg/day (G5), as well as in the silymarin-treated group (G6). Specifically, the steatosis scores in the *A. camphorata* powder-administered groups (G4 and G5) and the positive control group (G6) decreased by approximately 20% and 24%, respectively, compared with those in the negative control group (G2) (Table 6).

The inflammation score in the negative control group was significantly higher than that in the normal control group (*p* < 0.01). All *A. camphorata* powder-administered groups and the positive control group demonstrated a statistically significant decrease in the inflammation score compared to the negative control group (*p* < 0.01). Additionally, none of the *A. camphorata* powder-administered groups exhibited a statistically significant difference in inflammation scores compared to the positive control group (Table 6).

**Figure 4:**
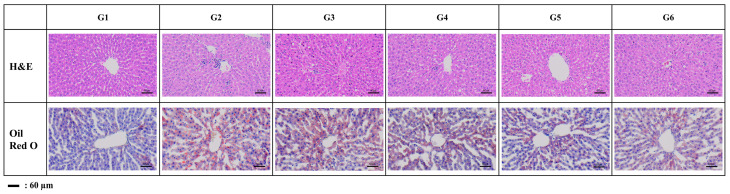
Representative histopathological profiles.

The ORO-positive area in the negative control group was significantly higher than that in the normal control group (*p* < 0.05). There was no significant difference in the ORO-positive area between the alcoholic fatty liver-induced groups (G2–G6). However, all *A. camphorata* powder-treated groups demonstrated a decrease of at least 21% in the ORO-positive area compared to the negative control group. Additionally, the positive control group showed a 25% decrease in the ORO-positive area compared to the negative control group (Figure 4 and Table 6).

## 4. Discussion

ALD, a potentially life-threatening condition akin to other chronic liver diseases, is the primary cause of chronic liver disease [1,3,7]. AFLD is the most common form of ALD [6,7]. Oxidative stress caused by ROS plays a vital role in ALD pathogenesis [6,7]. ROS, including H_2_O_2_, OH˙, and carbon-centered OH˙, are generated during ethanol metabolism [17,18]. ROS are closely related to the pathogenesis of hepatocellular injury as they suppress mitochondrial respiratory chain enzymes and deactivate glyceraldehyde-3-phosphate dehydrogenase as well as membrane sodium channels [19,20]. They also worsen inflammation and fibrosis by activating various signaling and transcription pathways [19,20]. They also increase lipid peroxidation, cytokine release, and lipid accumulation [1,17,18]. In this study, we evaluated the ROS-scavenging effect of *A. camphorata* powder by evaluating SOD expression levels in vitro and in vivo. Although the results of the SOD assays did not significantly differ in vitro or in vivo, we observed the potential effects of *A. camphorata* mycelium powder on SOD activity. In addition, GST activity was significantly increased in an *A. camphorata* powder dose-dependent manner compared to that in the negative control group. Earlier research has shown that both SOD and GST activities significantly increased in *A. camphorata*-treated mice, consistent with our results.

MDA is frequently utilized as a marker for lipid peroxidation, with elevated hepatic MDA levels being strongly associated with higher serum AST and ALT levels [12,21]. This is because higher ROS levels in the tissue result in higher lipid peroxidation and tissue damage, thus increasing MDA content. In the present study, lower MDA levels were observed in the *A. camphorata* mycelium powder-supplemented groups than in the corresponding controls. This may be attributed to high antioxidant enzyme activity and high antioxidant contents of *A. camphorata*.

**Figure 5:**
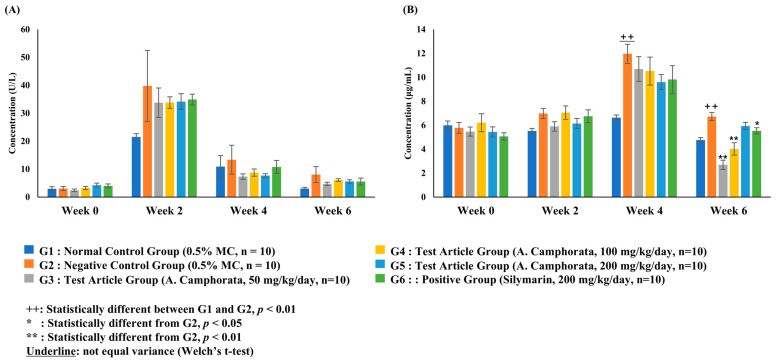
Concentration of γ-GTP (**A**) and adiponectin (**B**) in the rat serum. Data were presented as the mean ± SE. The results were statistically analyzed using Student’s *t*-test or one-way ANOVA.

ALT and AST levels are the major indicators of hepatocellular injury. Although serum levels of hepatic enzymes such as ALT and AST are not closely related to the severity of ALD, elevated levels of these enzymes are important markers of hepatic injury [22,23,24]. Patients with ALD have elevated AST, ALT, and ALP levels. In this study, the serum levels of AST, ALT, and ALP were significantly decreased in the G5 group (200 mg/kg *A. camphorata* mycelium powder administration group) compared to the negative control. In addition, the AST level in *A. camphorata* mycelium powder-treated groups was significantly lower than in the negative control. Moreover, we observed that the level of γ-GTP tends to decrease in an *A. camphorata* powder concentration-dependent manner. In contrast, adiponectin levels were significantly higher in the negative control group than in the normal control group. Several studies have reported that the adiponectin concentration is elevated in patients who chronically consume alcohol [25,26,27,28,29]. Elevated adiponectin concentrations may improve insulin sensitivity by activating intracellular signaling pathways such as AMP-activated protein kinase (AMPK), mTOR, nuclear transcription factor-κB (NF-κB), STAT3, and JNK [27,28,29]. However, the mechanisms underlying the increase in adiponectin levels associated with chronic alcohol consumption remain unclear. Therefore, comparing the changes in adiponectin concentration between the negative control group, *A. camphorata*-treated groups, and the positive control group may yield inconclusive results. The concentration of adiponectin in the *A. camphorata*-administered groups increased in a dose-dependent manner, as shown in Figure 5B. These results indicated that *A. camphorata* mycelium powder could potentially exert hepatoprotective effects. Notably, *A. camphorata* mycelium powder showed hepatoprotective properties similar to silymarin, a well-known liver protectant.

Prolonged alcohol intake leads to fatty liver and liver damage, which are associated with impaired cholesterol balance and marked by elevated hepatic cholesterol levels [30]. In this study, the negative control group showed a statistically significant increase in the levels of HDL, also known as good cholesterol, compared to the normal control group. However, a previous study reported that the level of dysfunctional HDL and the apoA transport rate were significantly associated with increased alcohol intake [31,32]. Therefore, it is more appropriate to focus on changes in CHO rather than HDL levels. Moreover, liver tissue analysis showed that the *A. camphorata* mycelium powder 100 mg/kg/day and 200 mg/kg/day groups showed a statistically significant decrease in liver TG levels compared to the negative control group.

Hypoglycemia is a common phenomenon in patients with alcoholic liver disease [33,34]. Previous studies have reported that alcoholic hypoglycemia is related to a decline in gluconeogenesis and/or an increase in insulin secretion [33,34]. Therefore, it is important to evaluate the pharmacological effects of *A. camphorata* mycelium powder on blood glucose levels. In this study, alcohol-induced low blood glucose levels were improved by the administration of *A. camphorata* mycelium powder at all doses on week 6.

Histopathological analysis showed that the *A. camphorata* mycelium powder administration group showed a statistically significant decrease in the inflammation score (*p* < 0.01) and a decreasing tendency in the steatosis score and ORO-positive area compared with the negative control group.

This study examines the efficacy of *A. camphorate* mycelium powder in addressing alcohol-induced hepatic damage, although it shows potential cytotoxic effects at high concentrations in vitro. Therefore, further studies need to be conducted to evaluate its toxicity.

## 5. Conclusions

In this study, we evaluated the efficacy of *A. camphorata* mycelium powder on ALD both in vitro and in vivo. We observed increased levels of antioxidative enzymes such as GSH and reduced levels of liver damage enzymes (ALT, AST, and ALP). Moreover, the levels of liver lipid metabolism (MDA and adiponectin) decreased in subjects supplemented with *A. camphorata* mycelium powder. Histopathological improvements were also observed. Taken together, *A. camphorata* mycelium powder may have beneficial effects in improving liver injury by reducing lipid peroxidation through the enhancement of antioxidative enzymes and lipid accumulation. These hepatoprotective effects are thought to be due to antroquinonol, the major constituent of *A. camphorata.* Especially, the antioxidant function of antroquinonol and its effect on activating the Nrf pathway to eliminate reactive oxygen species are thought to have a significant impact. Nonetheless, additional studies are necessary to comprehensively elucidate the mechanisms behind these effects.

## Figures and Tables

**Table 1:** Group identification.

Group	Sex	Age of Animals at 1st Dosing	No. of Animals	Dose (mg/kg/day)	Volume (mL/kg/day)	Treatment
G1	M	10 weeks	10	-	10	0.5% methyl cellulose
G2	M	10 weeks	10	-	10	0.5% methyl cellulose
G3	M	10 weeks	10	50	10	*A. camphorata* mycelium powder
G4	M	10 weeks	10	100	10	*A. camphorata* mycelium powder
G5	M	10 weeks	10	200	10	*A. camphorata* mycelium powder
G6	M	10 weeks	10	200	10	Silymarin

G1: Normal control group. G2: Negative control group. G2–G6: Alcoholic fatty liver-induced groups. G6: Positive control group.

**Table 2:** Results of the in vitro efficacy assays of *A. camphorata* mycelium powder at different concentrations.

Assay	Normal Control	Negative Control	Concentration of Test Substance (µg/mL)			Concentration of Positive Control (µg/mL)
			1 μg/mL	4 μg/mL	16 μg/mL	
SOD activity (%)	100 ± 0.0	90.6 ± 7.9	93.5 ± 1.4	142.1 ± 14.1	107.4 ± 10.2	170.5 ± 59.9
GST activity (%)	100 ± 0.0	82.8 ± 0.1 **	109.9 ± 2.9 ^$$^	99.5 ± 0.8 ^$$^	119.6 ± 4.1 ^$$^	108.2 ± 3.2 ^$$^
ALT concentration (pg/mL)	712.9 ± 7.8	728.1 ± 16.0	707.4 ± 8.5	679.8 ± 16.7	697.7 ± 8.9	674.3 ± 18.6
AST concentration (pg/mL)	155.1 ± 2.4	198.5 ± 11.8 **	181.1 ± 4.7 ^a^	171.5 ± 3.6 ^aa^	155.1 ± 2.4 ^aa^	157.0 ± 5.9 ^aa^
Oil Red O staining assay (OD values at 510 nm)	0.150 ± 0.009	0.221 ± 0.009 **	0.190 ± 0.001 ^$$^	0.159 ± 0.014 ^$$^	0.141 ± 0.009 ^$$^	0.044 ± 0.001 ^$$^

Data are presented as the mean ± SE. The results were statistically analyzed using Student’s *t*-test or one-way ANOVA. ^a^ *p* < 0.05, significant difference compared to the negative control; Dunnett’s *t*-test. ^aa^ *p* < 0.01, significant difference compared to the negative control; Dunnett’s *t*-test. ** *p* < 0.01, significant difference compared to the normal control; Student’s *t*-test. ^$$^ *p* < 0.01, significant difference compared to the negative control; Welch’s *t*-test.

**Table 3:** Liver weight and liver weight-to-body weight ratio measured in each group.

Group	Liver Weight (g)	Liver Weight-to-Body Weight Ratio (%)
G1 (*n* = 10)	8.90 ± 0.20	2.13 ± 0.03
G2 (*n* = 10)	9.67 ± 0.37	2.56 ± 0.05 ^++^
G3 (*n* = 10)	10.20 ± 0.34	2.65 ± 0.05
G4 (*n* = 10)	10.03 ± 0.23	2.65 ± 0.07
G5 (*n* = 10)	9.95 ± 0.26	2.60 ± 0.04
G6 (*n* = 10)	10.03 ± 0.29	2.64 ± 0.04

Data are presented as the mean ± SE. The results were statistically analyzed using Student’s *t*-test or one-way ANOVA. ++: statistically significantly different between the G1 and G2 groups, *p* < 0.01.

**Table 4:** Results of liver tissue assays using *A. camphorata* mycelium powder at different concentrations.

Group	MDA (nmol/g)	SOD (U/mg)	TG (nmol/mg)	ADH (ng/mg)	ALDH (ng/mg)
G1 (*n* = 10)	0.52 ± 0.05	6.25 ± 0.32	26.86 ± 0.40	30.78 ± 6.43	0.21 ± 0.02
G2 (*n* = 10)	1.40 ± 0.14 ^++^	6.42 ± 0.96	33.64 ± 1.42 ^++^	56.26 ± 12.91	0.19 ± 0.01
G3 (*n* = 10)	0.89 ± 0.07 **	5.50 ± 0.49	32.04 ± 1.51	55.73 ± 14.66	0.19 ± 0.01
G4 (*n* = 10)	0.84 ± 0.08 **	6.05 ± 0.38	27.91 ± 0.82 *	58.66 ± 12.91	0.19 ± 0.01
G5 (*n* = 10)	0.77 ± 0.07 **	6.14 ± 0.28	28.74 ± 0.72 *	59.12 ± 12.24	0.23 ± 0.03
G6 (*n* = 10)	1.25 ± 0.09	6.61 ± 0.59	26.94 ± 1.54 **	39.03 ± 8.48	0.23 ± 0.01

Data are presented as the mean ± SE. The results were statistically analyzed using Student’s *t*-test or one-way ANOVA. ++: statistically different between the G1 and G2 groups, *p* < 0.01. *: statistically significantly different compared to the G2 group, *p* < 0.05. **: statistically significantly different compared to the G2 group, *p* < 0.01. Underlined: unequal variance (Welch’s *t*-test or Dunnett’s post hoc test).

**Table 5:** Relative mRNA expression levels of FAS, CYP2E1, CB1, and ERR-γ.

Group	FAS	CYP2E1	CB1	ERRγ
G1 (*n* = 10)	1.00 ± 0.32	1.00 ± 0.29	1.00 ± 0.07	1.00 ± 0.04
G2 (*n* = 10)	1.14 ± 0.33	1.27 ± 0.46	0.96 ± 0.08	1.00 ± 0.05
G3 (*n* = 10)	1.04 ± 0.55	2.12 ± 1.69	1.03 ± 0.07	0.88 ± 0.06
G4 (*n* = 10)	0.73 ± 0.19	1.35 ± 0.49	0.89 ± 0.06	0.90 ± 0.07
G5 (*n* = 10)	2.97 ± 1.14	1.38 ± 0.53	0.81 ± 0.06	0.91 ± 0.07
G6 (*n* = 10)	1.91 ± 0.80	0.84 ± 0.17	0.86 ± 0.06	0.83 ± 0.07

Data are presented as the mean ± SE. The results were statistically analyzed using Student’s *t*-test or one-way ANOVA.

**Table 6:** Results of histopathological analysis after treatment with *A. camphorata* mycelium powder at different concentrations.

Group	Steatosis Score (Max = 3)	Inflammation Score (Max = 3)	Oil Red O-Positive Area (%)
G1 (*n* = 10)	0.34 ± 0.06	0.24 ± 0.04	0.21 ± 0.04
G2 (*n* = 10)	1.00 ± 0.12 ^++^	1.20 ± 0.11 ^++^	2.15 ± 0.60 ^+^
G3 (*n* = 10)	0.94 ± 0.14	0.78 ± 0.08 **	1.68 ± 0.27
G4 (*n* = 10)	0.80 ± 0.08	0.74 ± 0.08 **	1.66 ± 0.43
G5 (*n* = 10)	0.80 ± 0.09	0.64 ± 0.08 **	1.70 ± 0.23
G6 (*n* = 10)	0.76 ± 0.09	0.70 ± 0.10 **	1.61 ± 0.25

Data are presented as the mean ± SE. The results were statistically analyzed using Student’s *t*-test or one-way ANOVA. +: statistically significantly different between the G1 and G2 groups, *p* < 0.05. ++: statistically significantly different between the G1 and G2 groups, *p* < 0.01. **: statistically significantly different compared to the G2 group, *p* < 0.01. Underlined: unequal variance (Welch’s *t*-test or Dunnett’s post hoc test).

## Data Availability

The original contributions presented in the study are included in the article, further inquiries can be directed to the corresponding author.
